# Impact of Sensory Sensitivity on Physiological Stress Response and Novel Peer Interaction in Children with and without Autism Spectrum Disorder

**DOI:** 10.3389/fnins.2016.00278

**Published:** 2016-06-23

**Authors:** Blythe A. Corbett, Rachael A. Muscatello, Scott D. Blain

**Affiliations:** ^1^Department of Psychiatry and Behavioral Sciences, Vanderbilt UniversityNashville, TN, USA; ^2^Vanderbilt Brain Institute, Neuroscience Graduate Program, Vanderbilt UniversityNashville, TN, USA; ^3^Department of Psychology and Human Development, Vanderbilt UniversityNashville, TN, USA

**Keywords:** autism, stress, cortisol, sensory, peer interaction, social, auditory

## Abstract

**Background:** For many children with Autism Spectrum Disorder (ASD), social interactions can be stressful. Previous research shows that youth with ASD exhibit greater physiological stress response during peer interaction, compared to typically developing (TD) peers. Heightened sensory sensitivity may contribute to maladaptive patterns of stress and anxiety. The current study investigated between-group differences in stress response to peer interaction, as well as the role of sensory sensitivity.

**Methods:** Participants included 80 children (40 ASD) between 8 and 12 years. Children participated in the peer interaction paradigm (PIP), an ecologically valid protocol that simulates real-world social interaction. Salivary cortisol was collected before and after the 20 min PIP. Parents completed questionnaires pertaining to child stress (*Stress Survey Schedule*) and sensory sensitivity (*Short Sensory Profile*). Statistical analyses included *t*-tests and ANCOVA models to examine between-group differences in cortisol and play; Pearson correlations to determine relations between cortisol, play, and questionnaire scores; and moderation analyses to investigate interactions among variables.

**Results:** Controlling for baseline cortisol values, children with ASD showed significantly higher cortisol levels than TD peers, in response to the PIP [*F*_(1, 77)_ = 5.77, *p* = 0.02]. Cortisol during play was negatively correlated with scores on the SSP (*r* = −0.242, *p* = 0.03), and positively correlated with SSS (*r* = 0.273, *p* = 0.02) indicating that higher cortisol was associated with greater sensory sensitivity (lower SSP reflects more impairment) and enhanced stress in various contexts (higher SSS reflects more stress). Furthermore, diagnosis was a significant moderator of the relation between cortisol and SSP, at multiple time points during the PIP (*p* < 0.05).

**Conclusions:** The current study extends previous findings by showing that higher physiological arousal during play is associated with heightened sensory sensitivity and a pattern of increased stress in various contexts. Results are discussed in a broader context, emphasizing the need to examine relationships between social, behavioral, and physiological profiles in ASD to enhance understanding and improve treatments aimed at ameliorating stress and sensory dysfunction, while enhancing social skills.

## Introduction

Impaired social communication is a primary characteristic of Autism Spectrum Disorder (ASD) (American Psychiatric Association, 2013) and may be the result of limitations in social cognition (e.g., Baron-Cohen, 1995) or social motivation (Chevallier et al., 2012). Underlying physiological factors may also impact the extent to which individuals with ASD engage with others in social situations. For example, there is considerable evidence that social interactions are highly stressful for children with ASD (Lopata et al., 2008; Corbett et al., 2010). It may be this exaggerated stress response that drives, in part, reductions in social engagement. There is also significant prevalence of sensory dysfunction in ASD (Rogers et al., 2003; Kern et al., 2006; Tomchek and Dunn, 2007; Ben-Sasson et al., 2009), and these hyper- and/or hypo- sensory sensitivities may further contribute to atypical patterns of social interaction.

Compared to typically developing (TD) peers, children with ASD tend to interact less with other children (Corbett et al., 2010, 2012) and engage in less cooperative play (Corbett et al., 2014b), instead showing a preference to engage in self-play (Humphrey and Symes, 2011). There is also evidence that individuals with ASD have abnormal preferences for interpersonal distance (IPD), including both standing too close or preferring more distance relative to TD peers (Kennedy and Adolphs, 2014; Lough et al., 2015; Perry et al., 2015). As children with ASD become more aware of their limitations in social skills with age (Knott et al., 2006), later peer interactions may be characterized by exacerbated social stress (Schupp et al., 2013). As a result of this stress, these children may display increased avoidance behaviors characterized by decreased social motivation to interact with peers. This notion is supported by research showing that higher cortisol response to a novel social interaction was associated with reduced social communication among children with ASD (Corbett et al., 2014b).

The hypothalamic-pituitary-adrenal (HPA) axis is a highly regulated system that is responsible for maintaining homeostasis via maintenance of a diurnal rhythm, activation in response to stress or threat, and restoration of basal activity via negative feedback mechanisms. Cortisol, an important stress hormone, serves as a measurable indicator of HPA regulation, increasing in concentration after exposure to stressors (Herman and Cullinan, 1997). Cortisol also shows a diurnal rhythm of fluctuating concentration, throughout the day. Typically, diurnal cortisol values are at peak levels in the morning and slowly decline to lowest levels in the evening. In ASD, the diurnal rhythm appears to be altered, such that children with ASD have lower morning cortisol and higher evening cortisol values relative to TD children (Corbett et al., 2008, 2009). Further, many children with ASD show an increased response to stress, as indicated by higher cortisol levels, when interacting with unfamiliar peers (Lopata et al., 2008; Corbett et al., 2010). This stress response appears to be tied to level of social cognition, as those with highest cortisol values tend to show less social motivation (Corbett et al., 2014b). It is important to note, however, that this response is considerably variable within the ASD population (Lopata et al., 2008; Schupp et al., 2013). This suggests the possibility of interactions with other characteristics, which may explain part of the high variability in psychosocial stress response as seen in ASD.

Sensory processing abnormalities are now considered a core symptom of ASD under the Restrictive and Stereotypic Behaviors criteria of the DSM-5 (American Psychiatric Association, 2013). Sensory behaviors have historically been divided into four patterns, including sensory hypo-reactivity, hyper-reactivity, sensory seeking, and sensory avoidance (Dunn, 2001; Ben-Sasson et al., 2009). Complexity arises from the fact that many individuals with ASD will show several of these patterns at one time, with mixed patterns being displayed across sensory domains (Baranek et al., 2006; Tomchek and Dunn, 2007; Lidstone et al., 2014). Recent conceptualizations, therefore, have attempted to classify sensory processing abnormalities in ASD into specific subtypes (Lane et al., 2011, 2014; Uljarević et al., 2016), such as sensory adaptive, sensory moderate, and sensory severe (Uljarević et al., 2016).

Emerging research demonstrates a relationship between sensory sensitivities and arousal/anxiety in ASD. In one study, children with ASD showed lower morning cortisol relative to TD peers, which was associated with higher parent-reported stress as measured by the Stress Survey Schedule (SSS) (Corbett et al., 2009). Similarly, morning cortisol was associated with various domains of sensory functioning derived from the Short Sensory Profile—a parent-reported indicator of sensory sensitivities—(SSP; Dunn, 1999) painting a complex picture based on individual profiles (Corbett et al., 2009). Other studies investigating sensory profiles in ASD have also found relationships between sensory responsivity and anxiety (Green et al., 2012; Lidstone et al., 2014; Wigham et al., 2015; Uljarević et al., 2016), repetitive behaviors and stereotyped movements (Gabriels et al., 2008; Gal et al., 2010; Lidstone et al., 2014), and intolerance of uncertainty (Chamberlain et al., 2013; Wigham et al., 2015). Furthermore, a sample of TD young adults showed a correlation between sensory sensitivity and increased interpersonal distance (Perry et al., 2016), suggesting some role of sensory processing in social behavior. Currently, however, there is limited research that attempts to elucidate the impact of sensory dysfunction on social motivation and communication, especially in a naturalistic play setting.

The current study sought to investigate between-group differences in biobehavioral profiles of psychosocial stress following play with unfamiliar peers, in conjunction with sensory profiles. In order to elucidate the impact of sensory dysfunction on stress and social interaction, the current study sought to measure social engagement during a Peer Interaction Paradigm (PIP) (Corbett et al., 2010) and then to compare social behavior to stress and sensory profiles, as indicated by measures of cortisol and parent reports of stress and sensory sensitivity. It was hypothesized that children with ASD would have greater cortisol in response to play, relative to TD peers. Furthermore, it was hypothesized that cortisol response would be positively correlated with both increased parent-reported stress and sensory sensitivity.

## Materials and methods

### Participants

The sample included 80 un-medicated, pre-pubertal, children between the ages of 8-to 12 years, including 40 with ASD (mean = 9.65 years) and 40 TD (mean = 9.79 years). The gender composition included 14 females (6 = ASD, 8 = TD) and 66 males (34 = ASD, 32 = TD). ASD diagnosis was based on the Diagnostic and Statistical Manual (DSM-5) criteria (American Psychiatric Association, 2013) and established by all of the following: (1) a previous diagnosis by a psychologist, psychiatrist, or behavioral pediatrician with ASD expertise; (2) current clinical judgment; and (3) corroborated by the ADOS (Lord et al., 2000), administered by research-reliable personnel. For inclusion in the study all participants were required to have an estimated IQ of 80 or above, as measured by the Wechsler Abbreviated Scale of Intelligence (Wechsler, 1999). In addition, pubertal status was determined by the Pubertal Development Scale (Petersen et al., 1988) to confirm that the child had not yet entered puberty.

The Vanderbilt University Institutional Review Board approved the study. The investigation was performed in accordance with the Helsinki Declaration for research involving human subjects. Prior to inclusion in the study, informed written consent from parents and verbal assent from research participants were obtained. Participants were recruited by IRB approved flyers and several recruitment systems via clinics, subject tracking systems, resource centers, support groups, schools, and recreational facilities.

As described below, the study also included children who served as confederates (actor that participates in the study), who were of the same age and gender as the ASD and TD children. Parents of the confederates provided informed consent for them to train for and participate in the study. Confederates were selected based on demonstrated strong social skills, genuine desire to play and interact with children with and without disabilities, and an ability to follow research personnel instructions and translate them into age appropriate play behaviors. All confederates underwent several training procedures, including reading an instruction manual, direct skills modeling, and playground practice. The majority of the confederates had previously participated in research or served as a peer helper for children with disabilities.

### Peer interaction playground paradigm

The Peer Interaction Paradigm examines social exchanges between children with and without ASD within a naturalistic playground environment (Corbett et al., 2010). The playground is a 130 by 120 ft. fenced in play area that is part of the Susan Gray Preschool. The 20-min paradigm consisted of periods of free play and opportunities for cooperative play, which was facilitated by an age- and gender-match TD confederate. The trained confederate followed cues provided by research personnel through an earpiece with a remote transmitter, in order to provide structure to play by simultaneously soliciting play with both research participants. Use of a confederate permitted each interactive sequence of free or solicited play to occur within an otherwise natural setting, while also maintaining an even level of play to prevent increased aerobic activity, which could affect cortisol levels. In addition to the confederate, the paradigm involved one child with ASD and one TD child, all three of whom were unfamiliar with each other prior to the interaction. Each participant only took part in one 20-min session, such that the exposure represented a novel social experience. During the protocol, research personnel remained in the building in order to facilitate more natural play behavior.

**Figure 1:**
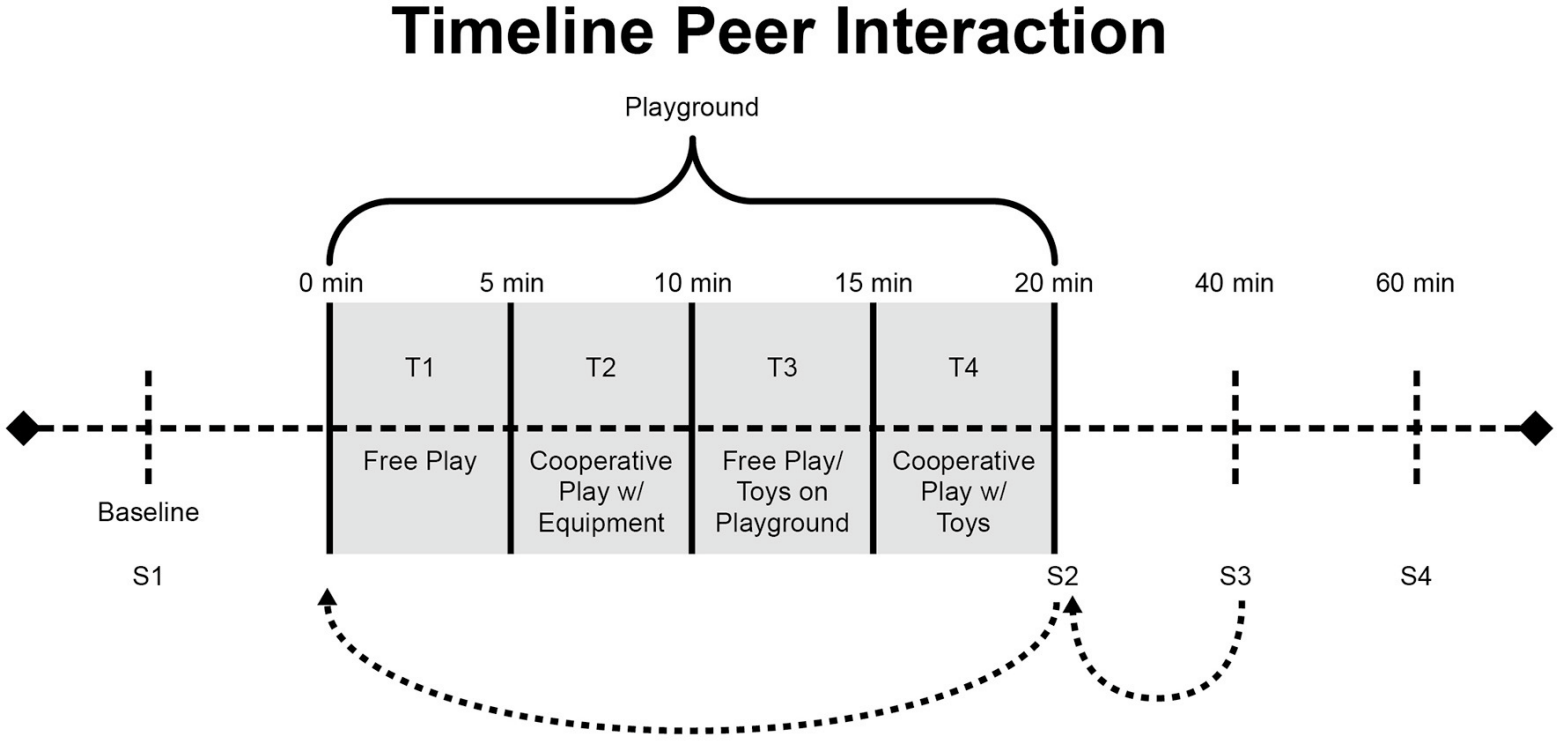
**Peer interaction paradigm timeline of events**. The 20-min paradigm consists of four 5-min periods of free play (T1), solicited play with equipment (T2), free, unsolicited play (T3), and solicited cooperative play with toys (T4). Salivary cortisol measures were taken at baseline (S1), immediately after the paradigm (S2), 20-min after the paradigm (S3), and 40-min after (S4).

The paradigm was divided into four 5-min time (T) periods of intermittent free play and solicited play (see Figure 1). The first period (T1) consisted of unsolicited free play. During the second period (T2), the confederate was instructed to solicit interaction for cooperative play on the playground equipment. During the third period (T3), the confederate returned to unsolicited free play. During the fourth period (T4), the confederate was instructed to once again solicit the two participants to engage in a cooperative game involving toys.

Interactions were recorded using state-of-the-art video equipment, including four professional Sony EVI D70 (Sony, New York, NY, USA) remotely operated cameras housed in glass cases, which were affixed to the four corners of the playground's fence. The cameras contain pan, tilt, and zoom features allowing full capture of the playground. To record remote audio communication, Motorola MC22OR GMRS-FRS (Motorola, Libertyville, IL, USA) and Audio-Technica (Audio-Technica, Stow, Ohio, USA) transmitters and receivers functioning as battery-operated microphones were clipped to each child's shirt. Audio was recorded using an eight-channel mixing board.

### Behavior coding

The Observer XT Version 8.0 software was used for the collection and analysis of the observational social interaction data (Noldus, 2008). Data were analyzed based on a predefined list of operationalized behaviors (Corbett et al., 2010; Schupp et al., 2013) by research-reliable raters who were unaware of the current study aims. Behaviors were analyzed using a transactional approach (i.e., *who does what to whom*) based on predefined operationalized behaviors (Mendoza and Mason, 1989; Lyons et al., 1990, 1992; Mason et al., 1993). Inter-rater reliability was calculated using Cohen's Kappa at *K* = 0.80, while test-retest reliability was *K* = 0.89. Cooperative play interactions were calculated as percentage of time engaged (verbal 90% and *K* = 0.85; group play 91% and *K* = 0.89). Variables such as cooperative play and verbal interaction were operationalized based upon previously described definitions and as part of a larger study (Corbett et al., 2014b). For the purpose of this study, cooperative play was defined as the percentage of time engaged in a reciprocal activity for enjoyment that involved and relied on the participation of two or more children (e.g., hide and seek). For an expanded description of the behavior coding protocol and operationalized variables, see previous studies (Corbett et al., 2010, 2014b; Schupp et al., 2013).

### Salivary cortisol sampling

Basal levels of salivary cortisol were collected from home to ascertain the child's afternoon baseline over 3 days, using established methods, as part of a larger study (Corbett et al., 2008). For the purpose of this study, only playground cortisol levels were analyzed.

It is important to note that there is an approximate 20-min time lag between when an event occurs and when a related change in cortisol can be detected in saliva (Kirschbaum and Hellhammer, 1989). The peer interaction included four salivary cortisol samples taken 20 min apart for each subject: S1–(baseline), S2–(immediately post-play), S3–(20 min post-play), and S4–(40 min post play). The S2 measurement taken immediately after the peer interaction represents circulating cortisol levels at the start of the paradigm, while the S3 measurement is representative of levels at the end of the peer interaction (see Figure 1). All peer interactions were held in the afternoon between 13:00 and 16:00, for comparison to afternoon baseline values.

Immediately before and following the playground paradigm, the ASD and TD participants were each assigned to an individual room and sat with a research assistant for cortisol sampling. A similar bag of toys and activities was provided to each child in order to maintain consistency in experience across all participants. Samples were collected following the standardized drool procedures outlined in previous studies (Corbett et al., 2008, 2010).

### Cortisol assay

The salivary cortisol assay was performed using a Coat-A-Count® radioimmunoassay kit (Siemens Medical Solutions Diagnostics, Los Angeles, CA) modified to accommodate lower levels of cortisol in human saliva relative to plasma. Saliva samples, which had been stored at −20°C, were thawed and centrifuged at 3460 rpm for 15 min to separate the aqueous component from mucins and other suspended particles. The coated tube from the kit was substituted with a glass tube into which 100 μl of saliva, 100 μl of cortisol antibody (courtesy of Wendell Nicholson, Vanderbilt University, Nashville, TN), and 100 μl of ^125^I-cortisol were mixed. After incubation at 4°C for 24 h 100 μl of normal rat serum in 0.1% PO4/EDTA buffer (1:50) and precipitating reagent (PR81) were added. The mixture was centrifuged at 3460 rpm for 30 min, decanted, and counted. Serial dilution of samples indicated a linearity of 0.99. Interassay coefficient of variation was 10.4%.

### Dependent measures

#### Short sensory profile (SSP; Dunn, 1999)

The SSP is a parent-report questionnaire designed to assess sensory sensitivity across seven sensory domains, such as auditory sensitivity, tactile sensitivity, and underresponsive/seeks sensation. Parents are asked to rate the frequency with which their child engages in behaviors related to sensory sensitivity in each domain. Possible scores range from 5 points to 1 point, ranging from “never responds in this manner” (5 points) to “always responds in this manner” (1 points). High raw scores indicate typical performance, while lower scores on the SSP are indicative of greater sensory dysfunction. The primary variable of interest for analysis was total raw score.

#### Stress survey schedule (SSS; Groden et al., 2001)

The SSS is a 60-item, parent-report survey designed to measure the daily stress of individuals with ASD and other developmental disabilities. The survey addresses eight domains of stress: Anticipation/Uncertainty, Changes and Threats, Unpleasant Events, Pleasant Events, Sensory/Personal Contact, Food Related Activity, Social/Environmental Interactions, and Ritual Related Stress. Parents are asked to rate intensity of stress for each item on a five-point Likert scale, ranging from none to mild stress (1) to severe stress (5). Internal consistency correlations range from 0.70 to 0.87. Higher total scores are indicative of enhanced stress, and total raw score was used for analysis.

#### Salivary cortisol

The primary outcome variable of interest for characterizing the stress response was salivary cortisol. Samples were analyzed for S1–S4, as described above. Because salivary cortisol measurements are positive and skewed toward large values, a log transformation was performed to achieve approximate normality. Log-transformed values were used in all analyses.

#### Social behavior

The primary outcome variable for social behavior was percent time spent in cooperative play during solicited play at T4 of the PIP.

### Statistical analysis

Descriptive statistics were calculated for demographic information, as well as parent-report measures of sensory functioning (SSP) and child stress (SSS); possible group differences in these variables were examined using independent samples *t*-tests. Analysis of covariance (ANCOVA) models were utilized to examine group differences in salivary cortisol response, controlling for baseline cortisol values. Pearson product moment correlations were conducted to examine associations between the sensory and stress measures. Moderation analyses were conducted to determine whether diagnosis was a moderator of the relation between SSP and cortisol response.

All statistical analyses were conducted using SPSS Version 22.0 (IBM Corp, 2013). The PROCESS application for SPSS was used to conduct moderation analyses (Hayes, 2013).

## Results

Demographic information for each group is presented in Table 1, including age, cognitive, and diagnostic information. Descriptive statistics for SSP, SSS, and cooperative play (separated by group) are listed in Table 2. Using independent samples *t*-tests, there were significant between-group differences on the SSP [*t*_(78)_ = 140.44, *p* < 0.0001] and the SSS [*t*_(77)_ = 109.32, *p* < 0.0001], showing greater parent-reported stress and sensory sensitivity in the ASD group, compared to the TD group.

**Table 1:** **Demographics**.

**Variable**	**ASD mean (SD)**	**TD mean (SD)**	***P*-value**
Age	9.65 (1.485)	9.79 (1.631)	0.699
SCQ total	20.72 (7.229)	2.37 (2.123)	<0.0001
Performance IQ	111.03 (53.394)	112.45 (12.831)	0.973
Verbal IQ	108.72 (62.858)	112.16 (13.997)	0.240
WASI estimated IQ	101.22 (22.505)	119.47 (13.123)	<0.001

*ASD, Autism spectrum disorder; IQ, intelligence quotient; TD, typical development; SCQ, social communication questionnaire; WASI, Weschler abbreviated scale of intelligence*.

**Table 2:** **Descriptive statistics for parent-report measures and play**.

**Variable**	**ASD mean (SD)**	**TD mean (SD)**	***P*-value**
**TOTAL SCORES**			
Short sensory profile	122.28 (21.393)	172.82 (16.629)	<0.0001
Stress survey schedule	122.02 (30.243)	62.59 (18.823)	<0.0001
**PERCENT TIME INTERACTING**			
Cooperative play	55.15 (36.08)	74.54 (24.13)	0.005

In order to assess group differences in social behavior, the percent duration of cooperative play during the solicited play period T4 of the PIP was compared between groups, using independent samples *t*-tests. Results revealed a significant difference between groups in percent duration of cooperative play [*t*_(83)_ = 8.349, *p* = 0.005], showing that children with ASD engage in significantly less cooperative play than TD peers.

Descriptive statistics for cortisol at baseline, during play, and after play are shown in Table 3. Analysis of covariance (ANCOVA) was conducted to examine the effect of group on stress (cortisol levels during cooperative play with peers), while controlling for baseline values. Results indicate a significant group difference in cortisol during play (S3) with peers [*F*_(1, 77)_ = 5.77, *p* = 0.02]. There was also a significant group difference in cortisol after play (S4) [*F*_(1, 77)_ = 4.78, *p* = 0.03].

**Table 3:** **Descriptive statistics for log cortisol at baseline, in response to the PIP, and after the PIP**.

**Cortisol value**	**ASD mean (SD)**	**TD mean (SD)**
Baseline cortisol (S1)	−0.1515 (0.333)	−0.1034 (0.267)
Cortisol during play (S3)	−0.1284 (0.303)	−0.2730 (0.373)
Cortisol after play (S4)	−0.2190 (0.293)	−0.3148 (0.266)

In consideration of these group differences, Pearson product correlations were calculated to assess the relations between physiological stress (cortisol), sensory functioning (SSP), and parent-report stress (SSS). Cortisol during play was negatively correlated with scores on the SSP (*r* = −0.24, *p* = 0.03) and positively correlated with scores on the SSS (*r* = 0.27, *p* = 0.02). Therefore, as cortisol levels increased during the PIP, SSP scores decreased (indicating greater sensory dysfunction) and global stress as measured by the SSS increased.

**Figure 2:**
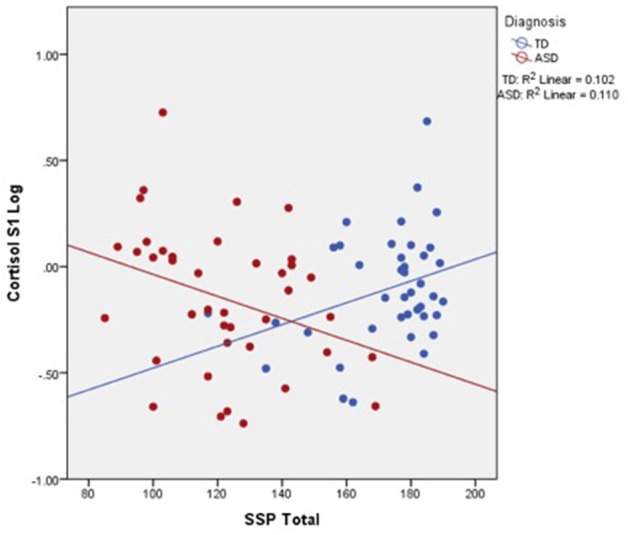
**Relationship between cortisol and sensory profile in children with ASD and TD peers**.

Moderation analysis was conducted to examine the impact of diagnosis on the relation between stress (cortisol) and sensory (SSP) functioning. Results show that diagnosis was a significant moderator of the association between SSP and cortisol at baseline (S1) [Δ*R*^2^ = 0.01, *F*_(1, 76)_ = 8.46, *p* = 0.0047], the beginning of play (S2) [Δ*R*^2^ = 0.06, *F*_(1, 76)_ = 4.85, *p* = 0.03], during play (S3) [Δ*R*^2^ = 0.02, *F*_(1, 76)_ = 3.94, *p* = 0.05], and after play (S4) [Δ*R*^2^ = 0.05, *F*_(1, 76)_ = 3.95, *p* = 0.05]. Figure 2 shows the moderating effect of diagnosis on the relation between SSP and cortisol, indicating that the negative correlation observed between cortisol and sensory functioning in the total sample is driven by the ASD group, whereas the opposite trend appears to hold true for the TD group.

In order to examine the impact of sensory sensitivity on play, we examined the association between SSP score and percent duration spent in cooperative play during the solicited play period, T4. There was a trend level positive association between cooperative play and total SSP (*r* = 0.197, *p* = 0.07). This association suggests that more sensory impairment was associated with less time spent in cooperative play (lower scores on the SSP are indicative of greater sensory dysfunction).

In an effort to further assess the impact of sensory sensitivity on play, *post-hoc* correlational analyses were conducted for individual domains of the SSP along with time spent in cooperative play. Scores on the Auditory domain were significantly positively correlated with cooperative play (*r* = 0.218, *p* = 0.04), while Underresponsive/Seeks Sensation correlated with play at a trend level (*r* = 0.196, *p* = 0.07).

## Discussion

While challenges in reciprocal social interaction are synonymous with ASD, sensory dysfunction and heightened stress are often found as well. Thus, the current study sought to examine the impact of sensory dysfunction on stress and social engagement with unfamiliar peers in children with and without ASD. The study utilized measures of social interaction (cooperative play) and physiological arousal (cortisol) during peer play using the PIP (Corbett et al., 2010) to assess the associations of social behavior and physiological stress with parent reports of stress and sensory sensitivity. It was hypothesized that children with ASD would show greater levels of salivary cortisol in response to play, relative to TD peers. Furthermore, it was hypothesized that cortisol response would be positively correlated with both increased parent-reported stress and sensory sensitivity. Therefore, the current study sought to further investigate biobehavioral profiles following play with unfamiliar peers with an emphasis on the impact of sensory sensitivity.

Autism is characterized by prevalence of sensory dysfunction (Rogers et al., 2003; Kern et al., 2006; Tomchek and Dunn, 2007; Ben-Sasson et al., 2009) and increased stress, especially during social interactions (Lopata et al., 2008; Corbett et al., 2010). In the current study, children with ASD showed both increased sensory dysfunction and parent-reported stress, relative to their TD peers. Research has shown that both altered sensory sensitivity and stress have deleterious effects on the overall functioning of children with ASD, especially given their potential associations with a variety of challenges, such as poor response to change (e.g., Chamberlain et al., 2013; Wigham et al., 2015) and anxiety (Green et al., 2012; Lidstone et al., 2014; Wigham et al., 2015; Uljarević et al., 2016).

In addition to group differences in sensory sensitivity and parent-reported stress, differences in salivary cortisol response were observed. Consistent with previous reports (Corbett et al., 2010, 2012, 2014b; Schupp et al., 2013), the ASD group had significantly greater physiological arousal in response to social interaction relative to the TD group, as indicated by higher cortisol levels both during and after the PIP. The increased arousal suggests that children with ASD may perceive social interaction with peers to be more stressful, compared to TD peers. Moreover, cortisol response was significantly correlated with parent-reported stress suggesting that heightened physiological reactions to peer interaction are also associated with a global pattern of elevated stress. Findings add to a growing body of work showing dysregulated patterns of stress in youth with ASD, both in terms of global and diurnal stress (Corbett et al., 2008, 2009), as well as stress in response to social interaction (e.g., Corbett et al., 2014b).

In light of observing group differences for both physiological stress and sensory sensitivity, we explored correlations between these variables. Significant associations were found between cortisol and SSP, such that higher cortisol in response to play was associated with greater sensory impairment. Moderation analyses were conducted to determine whether the correlation was driven by group status. Results showed that ASD diagnosis was a significant moderator of this relation, such that sensory dysfunction was associated with increased salivary cortisol response to peer interaction in the ASD group only. Moreover, this relationship held for cortisol levels at each time point of collection, including baseline cortisol as well as before, during, and after play. Interestingly, the TD group appeared to have the opposite relationship between cortisol and sensory functioning (Figure 2), implying that the negative impact of sensory sensitivity on stress reactivity during play is unique to ASD. In other words, greater levels of sensory dysfunction in ASD may lead to increased physiological stress. Thus, it appears that the symptom profile of ASD ostensibly contributes to enhanced reactivity to the environment, which can be manifested in both sensory and physiological functioning. The question still remains whether or not these relationships between stress and sensory sensitivity are bi-directional or whether a uni-directional model exists, such that one symptom precedes and drives the other.

When assessing group differences in cooperative play, the ASD group engaged in significantly less cooperative play, relative to the TD group. This is in line with previous findings that children with ASD tend to engage less than their TD peers during the PIP (e.g., Corbett et al., 2014b). While factors such as social cognition (Baron-Cohen, 1995; Corbett et al., 2014a) and social motivation (Chevallier et al., 2012; Corbett et al., 2014b) appear to play a significant role in explaining group differences in social engagement, sensory dysfunction may also have an impact on social behavior. For example, sensory sensitivity was recently shown to associate with interpersonal distance in TD individuals (Perry et al., 2016). In order to better understand the potential impact of sensory sensitivity on play behavior in ASD, duration spent in cooperative play was analyzed along with sensory processing scores. While overall sensory dysfunction was not associated with play behavior, auditory sensitivity was significantly correlated with play.

Adults with ASD have shown impairments in auditory localization through an inability to use prior perceptual experience to locate sounds (Skewes and Gebauer, 2016). Difficulties in auditory processing and orienting to a stimulus may be linked to emergence of impairments in social and communication behaviors early in development (Osterling et al., 2002). As such, children with ASD fail to orient to stimuli, especially critical social stimuli such as response to their own name (Dawson et al., 1998). It is possible that the inability to attend to stimuli is due, in part, to altered auditory processing, resulting in reduced early social interactions through a lack of joint attention with the caregiver. According to the Enactive Mind hypothesis (Klin et al., 2003), limited early social experiences may contribute to the social communication deficits that characterize ASD. Therefore, if sensory sensitivity alters one's experience in the physical world, interactions in the social world may be absent, incomplete, or unpredictable among children with ASD, thus leading to difficulties in social communication and cognition. While this is an intriguing theory for connecting the sensory and social symptoms of ASD, a great deal of empirical evidence is needed to test for direct links between sensory and social functioning in ASD.

### Limitations and future directions

The study had several strengths, including a relatively large sample size and use of a validated protocol for observing play behavior in a naturalistic playground setting. Some limitations do exist. Although parent-reports are the most commonly used measure of sensory profile in ASD and provide a useful representation of sensory dysfunction, future studies would benefit from more objective tests of sensory sensitivity such as assessment of various sensory domains (e.g., auditory, tactile) or through clinician-based observations. Furthermore, this study assessed only one novel exposure, representing everyday experiences of interacting with new people. A limitation of the current study is the lack of repeated exposure, which would allow for assessing stress response across several repeat social encounters. The study was also limited to one measure of physiological arousal (i.e., cortisol). Use of other measures such as respiratory sinus arrhythmia (e.g., Benevides and Lane, 2013) could provide a more detailed, continuous assessment of regulation and arousal throughout the protocol.

Novel social experiences are only one aspect of daily social interactions. The current study focused on these novel experiences. Results were consistent with previous reports (Corbett et al., 2010, 2012, 2014b; Schupp et al., 2013) suggesting that when exposed to new peers for the first time, children with ASD experience greater physiological stress and tend to engage in less cooperative play with those new people, relative to their TD peers. However, social interactions are not solely reliant on single interactions with new people. Children interact with the same peers on a daily basis at school, and physiological stress or sensory sensitivity could have significant effects on how children with ASD interact with familiar peers in a setting such as the classroom. Therefore, future studies should investigate the role of sensory sensitivity and physiological stress during repeat exposure.

The findings that sensory dysfunction may negatively impact social behavior is notable and warrants further investigation. It would be helpful to discern whether targeting sensory symptoms via treatment could indirectly improve social motivation in individuals with ASD. While results show several interactions are present between physiological stress, sensory sensitivity, and parent-report stress, the directionality among these variables remains unclear. Future studies are needed to determine the extent to which change in one variable leads to change in another. The findings that the association between cortisol and sensory sensitivity was driven by the ASD group requires further investigation to enhance understanding of the mechanisms behind the purported link, which appears specific to ASD. Finally, while auditory sensitivity and cooperative play were correlated, further research is needed to determine any possible causal impact of sensory sensitivity on social motivation.

### Summary

The primary objective of the current study was to examine the impact of sensory sensitivity on stress response and peer interaction in children with and without ASD. Results show that cortisol response to play is elevated in children with ASD and that higher physiological arousal during play is associated with greater sensory sensitivity and parent-reported stress. Additionally, ASD diagnosis confers risk for atypical sensory and stress reactivity, which may further contribute to the core social deficits. Taken together, results provide evidence for important interactions among social, sensory, and physiological profiles in ASD. Characterizing and assessing such profiles may better predict global outcomes, while also serving to inform more effective methods for intervention.

## Author contributions

The named authors made significant contributions to the investigation and manuscript. Specifically, BC conceptualized the study design, outlined the organization of the manuscript, guided the literature search, ran data analysis, provided interpretation to the findings, and made significant contributions to the final manuscript. RM read the extant literature, synthesized the results, contributed to interpretation of the findings, and co-wrote the initial draft of the manuscript. SB participated in the acquisition, analysis, and interpretation of the physiological and behavioral data, and contributed to the writing of the manuscript. All authors read and approved the content of the work.

## Funding

This work was supported by National Institute of Mental Health R01 MH085717 awarded to BC.

### Conflict of interest statement

The authors declare that the research was conducted in the absence of any commercial or financial relationships that could be construed as a potential conflict of interest.
